# Supplementary material for: Mechanisms governing the pioneering and redistribution capabilities of the non-classical pioneer PU.1
Source: Nat Commun. 2020 Jan 21;11:402. doi: 10.1038/s41467-019-13960-2 (PMC6972792; doi:10.1038/s41467-019-13960-2)
Supplement: Supplementary file 7 — Source data [file 41467_2019_13960_MOESM7_ESM.zip › Source_Data/Figure6/Figure6B_MotifScanOutput/FLI1_mutPU.1/homerResults/motif36.similar.html]

motif36

## Information for motif36

A
T
G
C
C
T
G
A
G
A
C
T
G
C
A
T
G
T
C
A
T
C
G
A
G
A
C
T
A
G
T
C
C
T
G
A
  
Reverse Opposite:  

G
A
C
T
T
C
A
G
C
T
G
A
A
G
C
T
C
A
G
T
C
G
T
A
C
T
G
A
G
A
C
T
T
A
C
G
  

|  |  |
| --- | --- |
| p-value: | 1e-28 |
| log p-value: | -6.521e+01 |
| Information Content per bp: | 1.558 |
| Number of Target Sequences with motif | 270.0 |
| Percentage of Target Sequences with motif | 2.63% |
| Number of Background Sequences with motif | 404.5 |
| Percentage of Background Sequences with motif | 1.06% |
| Average Position of motif in Targets | 98.4 +/- 57.6bp |
| Average Position of motif in Background | 104.5 +/- 57.4bp |
| Strand Bias (log2 ratio + to - strand density) | 0.1 |
| Multiplicity (# of sites on avg that occur together) | 1.02 |
| Motif File: | file (matrix) reverse opposite |

### Similar de novo motifs found

|  |  |  |  |  |  |  |  |
| --- | --- | --- | --- | --- | --- | --- | --- |
| Rank | Match Score | Redundant Motif | P-value | log P-value | % of Targets | % of Background | Motif file |
| 1 | 0.844 | C G A T A T C G T G C A A G T C G C A T C T G A G T C A C G A T T C A G G T C A A T G C | 1e-26 | -62.078170 | 2.06% | 0.74% | motif file (matrix) |
| 2 | 0.818 | A C G T C A T G C G T A A T G C G C A T C G T A G T C A A C G T A C T G G T C A | 1e-19 | -45.414028 | 1.06% | 0.29% | motif file (matrix) |
